# Dual tracer evaluation of dynamic changes in intratumoral hypoxic and proliferative states after radiotherapy of human head and neck cancer xenografts using radiolabeled FMISO and FLT

**DOI:** 10.1186/1471-2407-14-692

**Published:** 2014-09-22

**Authors:** Chowdhury Nusrat Fatema, Songji Zhao, Yan Zhao, Wenwen Yu, Ken-ichi Nishijima, Koichi Yasuda, Yoshimasa Kitagawa, Nagara Tamaki, Yuji Kuge

**Affiliations:** Department of Tracer Kinetics & Bioanalysis, Graduate School of Medicine, Hokkaido University, Kita 15, Nishi 7, Kita-ku, Sapporo, 060-8638 Japan; Department of Molecular Imaging, Graduate School of Medicine, Hokkaido University, Sapporo, Japan; Department of Nuclear Medicine, Graduate School of Medicine, Hokkaido University, Sapporo, Japan; Department of Oral Diagnosis and Medicine, Graduate School of Dental Medicine, Hokkaido University, Sapporo, Japan; Department of Integrated Molecular Imaging, Graduate School of Medicine, Hokkaido University, Sapporo, Japan; Central Institute of Isotope Science, Hokkaido University, Sapporo, Japan; Department of Radiation Medicine, Graduate School of Medicine, Hokkaido University, Sapporo, Japan

**Keywords:** Fluoromisonidazole, Fluorothymidine, Radiotherapy, Tumor reoxygenation and proliferation, Head and neck cancer xenograft

## Abstract

**Background:**

Radiotherapy is an important treatment strategy for head and neck cancers. Tumor hypoxia and repopulation adversely affect the radiotherapy outcome. Accordingly, fractionated radiotherapy with dose escalation or altered fractionation schedule is used to prevent hypoxia and repopulation. ^18^F-fluoromisonidazole (FMISO) and ^18^F-fluorothymidine (FLT) are noninvasive markers for assessing tumor hypoxia and proliferation, respectively. Thus, we evaluated the dynamic changes in intratumoral hypoxic and proliferative states following radiotherapy using the dual tracers of ^18^F-FMISO and ^3^H-FLT, and further verified the results by immunohistochemical staining of pimonidazole (a hypoxia marker) and Ki-67 (a proliferation marker) in human head and neck cancer xenografts (FaDu).

**Methods:**

FaDu xenografts were established in nude mice and assigned to the non-radiation-treated control and two radiation-treated groups (10- and 20-Gy). Tumor volume was measured daily. Mice were sacrificed 6, 24, and 48 hrs and 7 days after radiotherapy. ^18^F-FMISO, and ^3^H-FLT and pimonidazole were injected intravenously 4 and 2 hrs before sacrifice, respectively. Intratumoral ^18^F-FMISO and ^3^H-FLT levels were assessed by autoradiography. Pimonidazole and Ki-67 immunohistochemistries were performed.

**Results:**

In radiation-treated mice, tumor growth was significantly suppressed compared with the control group, but the tumor volume in these mice gradually increased with time. Visual inspection showed that intratumoral ^18^F-FMISO and ^3^H-FLT distribution patterns were markedly different. Intratumoral ^18^F-FMISO level did not show significant changes after radiotherapy among the non-radiation-treated control and radiation-treated groups, whereas ^3^H-FLT level markedly decreased to 59 and 45% of the non-radiation-treated control at 6 hrs (*p* < 0.0001) and then gradually increased with time in the 10- and 20-Gy-radiation-treated groups. The pimonidazole-positive hypoxic areas were visually similar in both the non-radiation-treated control and radiation-treated groups. No significant differences were observed in the percentage of pimonidazole-positive cells and Ki-67 index.

**Conclusion:**

Intratumoral ^18^F-FMISO level did not change until 7 days, whereas ^3^H-FLT level markedly decreased at 6 hrs and then gradually increased with time after a single dose of radiotherapy. The concomitant monitoring of dynamic changes in tumor hypoxia and proliferation may provide important information for a better understanding of tumor biology after radiotherapy and for radiotherapy planning, including dose escalation and altered fractionation schedules.

## Background

Over the past few decades, the preferred treatment strategies for squamous cell carcinoma of the head and neck have gradually shifted from surgery to organ-preservation approaches such as radiotherapy with or without chemotherapy. Tumor hypoxia and accelerated tumor proliferation are important factors that adversely affect the efficacy of radiotherapy [1–3]. Accordingly, in recent radiotherapy strategies, dose escalation and altered fractionation schedules are widely applied to prevent tumor hypoxia and accelerated tumor proliferation [2]. Head and neck tumors are highly hypoxic in nature and also show accelerated proliferation of clonogenic tumor cells during the course of treatment, which hinders the efficacy of radiotherapy and leads to locoregional treatment failure [4, 5]. Particularly in the case of moderately or poorly radiosensitive cancers, how they respond to radiotherapy remains a clinical dilemma for medical professionals. Sometimes there is no good outcome even after an entire session of radiotherapy.

Better understanding of the tumor microenvironment such as the tumor hypoxic state and proliferative activity allows a better optimal individualized plan for radiotherapy (such as dose escalation or altered fractionation schedule). For this purpose, numerous immunohistochemical or immunofluorescence microscopy techniques have been established. However, biopsy or excised tissue samples are required for all these techniques, and collection of such samples is invasive and prone to sampling error. Recently, the exploration of tumor hypoxic and proliferative states has shifted to diagnostic imaging owing to the rapidly developing field of molecular imaging. Specific biomarker probes can be administered and visualized in vivo with the aid of functional imaging modalities such as PET. ^18^F-Fluoromisonidazole (FMISO) and ^18^F-fluorothymidine (FLT) are widely used for noninvasive imaging of tumor hypoxia and tumor proliferation, respectively [6–13]. FMISO is reduced and trapped in hypoxic cells, and reflects tumor hypoxia. The proliferation marker FLT reflects thymidine kinase 1 (TK1) activity, which is extremely sensitive to ionizing radiation, and changes in FLT uptake level directly reflect the biological effect of radiation therapy [14, 15].

Intratumoral hypoxia or proliferative activity following radiotherapy was evaluated in several studies using FMISO or FLT [7–13]. There have been no studies, however, in which the dynamic changes in intratumoral hypoxic state and proliferative activity after single-dose radiotherapy were simultaneously evaluated using the dual tracers of FMISO and FLT. Such investigations may provide important information for a better understanding of tumor biology after radiotherapy and for radiotherapy planning, including dose escalation and altered fractionation schedules, particularly in moderately or poorly radiosensitive head and neck carcinoma. Thus, in this study, we simultaneously evaluated the dynamic changes in intratumoral hypoxic and proliferative states using FMISO and FLT, and further verified the results by immunohistochemical staining of pimonidazole and Ki-67 after a single dose of radiation of human head and neck cancer xenografts using a moderately radiosensitive cancer cell line, FaDu.

## Methods

### Radiopharmaceuticals

^18^F-FMISO was obtained from the Hokkaido University Hospital Cyclotron Facility, which was synthesized as previously described [16–18]. [Methyl-^3^H (N)]-3′-fluoro 3′-deoxythymidine (^3^H-FLT) (specific activity, 74–370 GBq/mmol) was purchased from Moravek Biochemicals Inc., CA.

### Xenograft model and irradiation

All experimental protocols were approved by the Laboratory Animal Care and Use Committee of Hokkaido University (approval number 13–0057) and were performed according to the Guidelines for Animal Experiments at the Graduate School of Medicine, Hokkaido University. Nine-week-old male BALB/c athymic nude mice (supplied by Japan SLC, Inc., Hamamatsu, Japan) were used in all experiments. A human head and neck cancer xenograft model was established in mice using the human head and neck squamous cell carcinoma cell line FaDu. FaDu cells (5 × 10^6^ cells/0.1 ml) were inoculated subcutaneously into the right flank of each mouse, as previously described [12].

**Figure 1:**
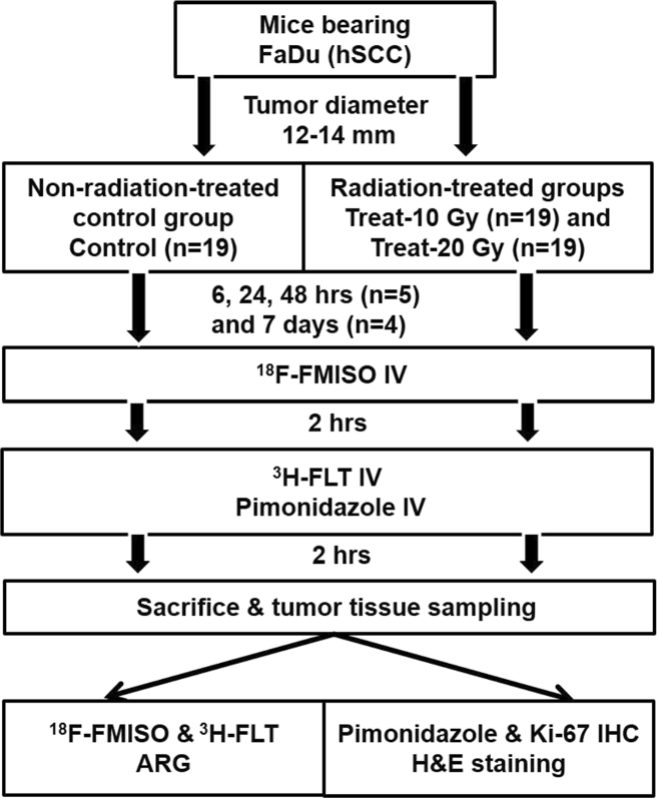
**Experimental protocol of this study.**

When the tumors reached 12–14 mm in diameter, the mice were randomly assigned to three groups: one non-radiation-treated control group (Control) and two radiation-treated groups at 10 and 20 Gy (Treat-10 Gy and Treat-20 Gy) (n = 19 for each group) (Figure 1). Data were collected at four time points for each group (6, 24, and 48 hrs, and 7 days from irradiation to killing; n = 5 for the time points of 6, 24, and 48 hrs and n = 4 for the time points of 7 days). A tumor growth curve was derived from the animals assigned to day 7 in each group. Tumor size was measured every day from the start of the radiation treatment and tumor volume was calculated using the following formula: π/6 × largest diameter × (smallest diameter)^2^.

In the radiation-treated groups, tumors were irradiated at a single dose of 10 or 20 Gy using an X-ray generator (MBR- 1520R-3; Hitachi, Tokyo, Japan) at a dose rate of 1.69 Gy/min, as previously described [12].

### Dual tracer (^18^F-FMISO and ^3^H-FLT) ARG study

Two hours after ^18^F-FMISO (18.5 MBq) injection, the mice were intravenously injected with ^3^H-FLT (0.185 MBq) and pimonidazole (a hypoxia marker) (60 mg/kg body weight). Two hours after ^3^H-FLT and pimonidazole injections, these mice were sacrificed and the tumors were excised. The excised tumors were then embedded in Tissue-Tek medium (Sakura Finetechnical Co., Ltd., Tokyo, Japan) with a calf muscle section and frozen in isopentane/dry ice. Serial cross sections of 10 μm (for autoradiographic exposure) or 5 μm (immunohistochemical staining) thickness were immediately cut and thaw-mounted on a glass slide.

The distribution of each tracer in the tumor tissue was determined by dual-tracer ARG [19]. Briefly, the same cryostat cross sections were exposed twice to phosphor imaging plates (Fuji Imaging Plate BAS-SR 2025 for ^18^F and BAS-TR 2025 for ^3^H, Fuji Photo Film Co., Ltd., Japan) together with a set of calibrated standards [20]. The first autoradiographic exposure was performed overnight to detect the radioactivity of ^18^F-FMISO. To allow the decay of ^18^F activity, the second exposure was started 1 week later to visualize the distribution of ^3^H-FLT. The second exposure required 5 weeks. After each exposure, the imaging plates were scanned using a Fuji Bio-imaging Analyzer FLA-7000 (Fuji Photo Film Co., Ltd., Minato-ku, Tokyo, Japan), and the images obtained were analyzed using image analysis software Multi Gauge (Ver. 3.0, Fuji Photo Film Co., Ltd., Japan).

For the quantitative evaluation of ^18^F-FMISO and ^3^H-FLT radioactivities, ROIs were placed to cover the entire tumor tissue and muscles on each ARG image with reference to the H&E sections. ^18^F-FMISO or ^3^H-FLT radioactivity in each ROI was separately recorded and calculated as percentage injected dose per gram (%ID/g) and normalized with body weight [(%ID/g) × kg], as previously described [12, 20, 21].

### Immunohistochemical staining

Frozen sections of the tumor were stained for pimonidazole (a hypoxia marker) and Ki-67 (a proliferation marker) to assess hypoxia and proliferation, respectively. The immunohistochemical staining of pimonidazole (Hypoxyprobe^TM-1^, HIP Inc.) was performed using a monoclonal antibody (rabbit IgG1, HIP Inc.) and that of Ki-67 was performed using a monoclonal rabbit anti-human Ki-67 antibody (clone SP6) (Thermo Fisher Scientific, CA, USA). For the quantitative analysis of hypoxia, the percentage of the area positively stained by the anti-pimonidazole antibody in the entire tumor cross section was calculated as the hypoxic fraction (%pimonidazole-positive area) using Image J. For the quantitative analysis of tumor proliferation, the Ki-67 labeling index, that is, the percentage of the number of Ki-67-positive nuclei to the total number of nuclei, was used. The number of Ki-67-positive nuclei and the total number of nuclei were counted under a microscope field (x400 magnification, 0.644 mm^2^ per field) using Image J. H&E staining was also performed.

### Statistical analyses

All statistical analyses were performed using Stat View version 5.0 (SAS Institute, Inc.). All values are expressed as mean ± SD (standard deviation). One-factor repeated measures analysis of variance (ANOVA) was carried out to compare tumor volume among the non-radiation-treated control and radiation-treated (10 and 20 Gy) groups. Two-way factorial ANOVA was performed to compare intratumoral ^18^F-FMISO level, intratumoral ^3^H-FLT uptake level, hypoxic fraction and percentage of Ki-67 labeling index with time among the three groups. One-way ANOVA followed by a Bonferroni post-hoc test was used to assess the significance of difference among the three groups at each time point and among the time points in each group for tumor volume and intratumoral ^3^H-FLT uptake level. Linear regression analysis was performed to determine the correlation between the tracer uptake levels of ^18^F-FMISO and ^3^H-FLT. A *p* value of < 0.05 was considered statistically significant.

**Figure 2:**
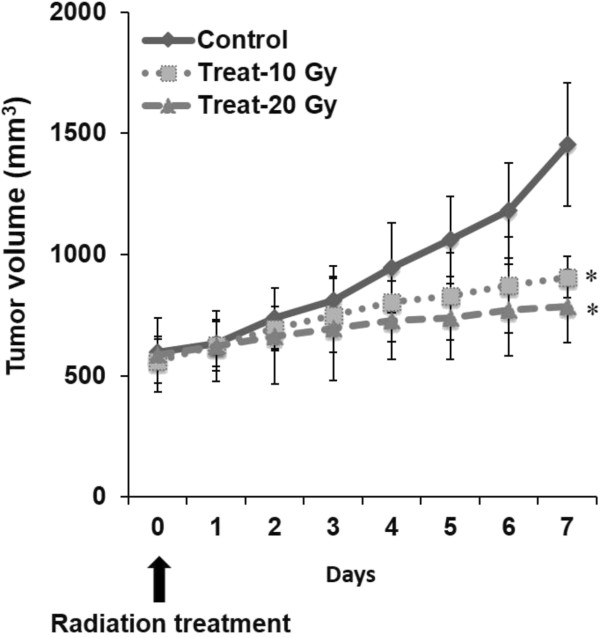
**Growth curves of tumors in non-radiation-treated control and 10- and 20-Gy radiation-treated mice.** Data are expressed as mean ± SD. *p < 0.01 for both Control vs Treat-10 Gy and Control vs Treat-20 Gy.

## Results

### Tumor volume change

The changes in tumor volume are shown in Figure 2. In the non-radiation-treated control group, tumor volume increased with time. Compared with the control group, the tumor growth was significantly suppressed in mice treated with 10 and 20 Gy radiation, but the tumor volume in these mice gradually increased with time. A significant difference in tumor volume was observed between the non-radiation-treated control and the radiation-treated groups on day 7 (*p* < 0.01 for both Control vs Treat-10 Gy and Control vs Treat-20 Gy), whereas no statistically significant difference was observed between the two radiation-treated groups. No significant differences in tumor volume were noted among the three groups until day 6.

### Visual and quantitative analysis of ^18^F-FMISO and ^3^H-FLT ARG image

**Figure 3:**
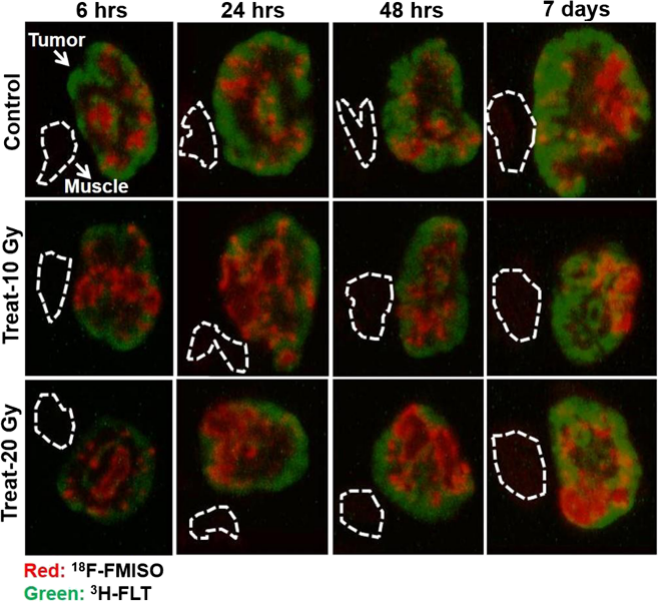
**Representative merged images of** ^**18**^ **F-FMISO and** ^**3**^ **H-FLT ARG.** Red represents ^18^F-FMISO distribution and green represents ^3^H-FLT distribution. The dotted line represents the outline of muscle tissue.

Figure 3 shows the representative merged images of ^18^F-FMISO and ^3^H-FLT ARG. In the non-radiation-treated control group, ^18^F-FMISO was distributed mostly in the central part of the tumor tissue at all time points. The 10- and 20-Gy-radiation-treated groups also showed a similar ^18^F-FMISO distribution pattern to the control group (Figure 3). On the other hand, ^3^H-FLT was distributed mostly in the peripheral part of the tumor tissue in both the non-radiation-treated control and radiation-treated groups, and ^3^H-FLT uptake level was low at 6 hrs compared with the control group, and then gradually increased with time, 24 and 48 hrs, and day 7 after 10 and 20 Gy radiation treatment (Figure 3).

**Figure 4:**
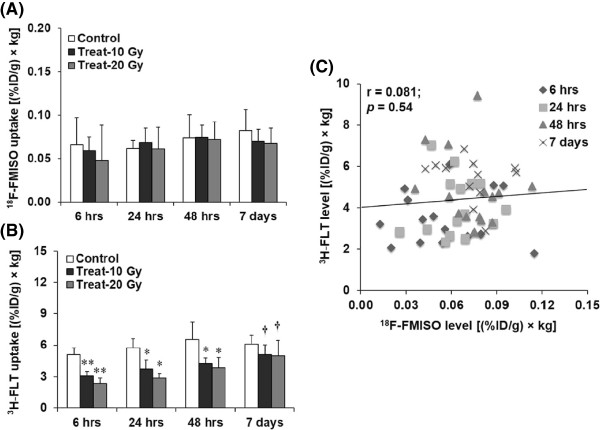
**Quantitative analysis of intratumoral (A)** ^**18**^ **F-FMISO and (B)** ^**3**^ **H-FLT uptake levels by ARG and (C) regression analysis of the uptake levels of** ^**18**^ **F-FMISO and** ^**3**^ **H-FLT.** Data are expressed as mean ± SD. *p < 0.01 and **p < 0.0001 for both Control vs Treat-10 Gy and Control vs Treat-20 Gy. †*p* < 0.001, 6 hrs vs day 7 for each group radiation-treated with 10 and 20 Gy.

Figure 4 shows the quantitative analysis of intratumoral ^18^F-FMISO and ^3^H-FLT uptake level and their correlation. In the non-radiation-treated control group, the ^18^F-FMISO level slightly increased with time, but the differences were not significant. No significant differences were observed among the non-radiation-treated control group and the radiation-treated groups at any time points. The ^18^F-FMISO levels in the tumors were 0.06 ± 0.03, 0.05 ± 0.01, and 0.04 ± 0.04 [(%ID/g) × kg] at 6 hrs, 0.06 ± 0.01, 0.06 ± 0.01, and 0.06 ± 0.025 [(%ID/g) × kg] at 24 hrs, 0.07 ± 0.02, 0.07 ± 0.01, and 0.07 ± 0.02 [(%ID/g) × kg] at 48 hrs, and 0.08 ± 0.02, 0.07 ± 0.01, and 0.06 ± 0.01 [(%ID/g) × kg] on day 7 in the non-radiation-treated control and the 10- and 20-Gy-radiation-treated groups, respectively (Figure 4A). On the other hand, intratumoral ^3^H-FLT uptake level significantly decreased to 59 and 45% (*p* < 0.0001) at 6 hrs in the 10- and 20-Gy-radiation-treated groups compared with the non-radiation-treated control group. Thereafter, intratumoral ^3^H-FLT uptake level gradually increased with time in both the radiation-treated groups, and the ^3^H-FLT uptake levels on day 7 were significantly higher than those at 6 hrs in the corresponding radiation-treated groups. The intratumoral ^3^H-FLT uptake levels were 64 and 50% at 24 hrs (*p* < 0.01 for both), 64 and 59% at 48 hrs (*p* < 0.01 for both), and 81 and 81% on day 7 (*p* = NS for both) in the mice treated at 10 and 20 Gy, respectively, compared with the non-radiation-treated control mice (Figure 4B). The intratumoral ^3^H-FLT uptake levels in the tumors were 5.11 ± 0.62, 3.06 ± 0.42, and 2.34 ± 0.52 [(%ID/g) × kg] at 6 hrs, 5.72 ± 0.90, 3.70 ± 0.88, and 2.86 ± 0.42 [(%ID/g) × kg] at 24 hrs, 6.56 ± 1.63, 4.22 ± 0.53, and 3.84 ± 0.94 [(%ID/g) × kg] at 48 hrs, and 6.11 ± 0.86, 5.08 ± 0.95, and 4.99 ± 1.48 [(%ID/g) × kg] on day 7 in the non-radiation-treated control and the 10- and 20-Gy-radiation-treated groups, respectively (Figure 4B). The relationship between ^18^F-FMISO and ^3^H-FLT uptake levels is shown in Figure 4C. No significant correlation was observed between the two tracer distributions (*r* = 0.081, *p* = 0.54).

### Immunohistochemical staining

**Figure 5:**
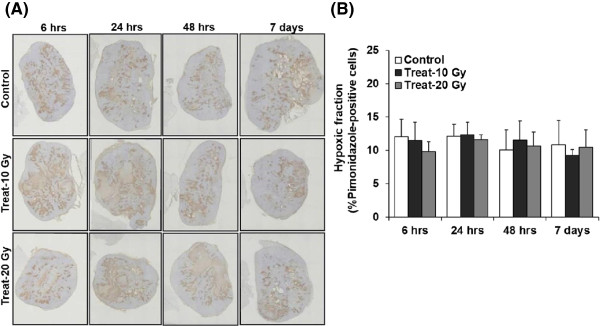
**Microscopy images and quantitative analysis of hypoxia. (A)** Microscopy images and **(B)** quantitative analysis of hypoxia. **(A)** Representative images of pimonidazole staining (brown staining in pimonidazole-positive cells). **(B)** Mean hypoxic fraction ± SD.

Representative images and the quantitative analysis of hypoxia (%pimonidazole-positive cells) by pimonidazole IHC are shown in Figure 5. The pimonidazole-positive hypoxic areas were visually similar in both the non-radiation-treated control and radiation-treated groups (Figure 5A). The hypoxic fractions in tumors were 12.0 ± 2.6, 11.6 ± 2.7, and 9.8 ± 1.4% at 6 hrs, 12.1 ± 1.8, 12.3 ± 1.8, and 11.6 ± 0.77% at 24 hrs, 10.07 ± 3.0, 11.49 ± 3.0, and 10.65 ± 2.1% at 48 hrs, and 10.8 ± 3.6, 9.23 ± 0.86, and 10.46 ± 2.6% on day 7 in the non-radiation-treated control and the 10- and 20-Gy radiation-treated groups, respectively (Figure 5B). No significant differences were observed in the percentage of pimonidazole-positive cells among the three groups (*F* = 0.36; *p* = 0.69) at various time points (*F* = 1.43; *p* = 0.24).

**Figure 6:**
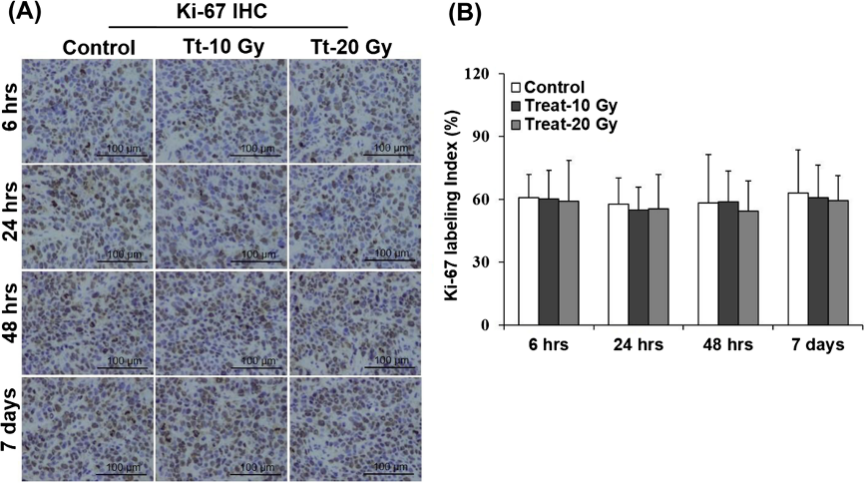
**Microscopy images and quantitative analysis of Ki-67 labeling index. (A)** Microscopy images and **(B)** quantitative analysis of Ki-67 labeling index. **(A)** Representative images of Ki-67 staining (brown staining in Ki-67 positive cells). **(B)** Mean Ki-67 labeling index ± SD.

Figure 6 shows the representative images and the quantitative evaluation of Ki-67 labeling index by IHC. No significant differences were observed in the percentage of Ki-67 labeling index with time and among the three groups (Figure 6A). The Ki-67 labeling indices in tumor tissues were 60.7 ± 10.8, 60.3 ± 13.4, and 59.1 ± 19.4% at 6 hrs, 57.7 ± 12.3, 54.8 ± 10.9, and 55.5 ± 16.2% at 24 hrs, 58.2 ± 23.1, 58.7 ± 14.6, and 54.2 ± 14.3% at 48 hrs, and 63.0 ± 20.4, 60.6 ± 15.6, and 59.3 ± 11.9% on day 7 in the non-radiation-treated control and the 10- and 20-Gy radiation-treated groups, respectively (Figure 6B).

## Discussion

The major findings of this dual-tracer study are as follows. Intratumoral ^18^F-FMISO level did not show any significant dynamic changes with time after single-dose radiotherapy among the non-radiation-treated control and radiation-treated groups. On the other hand, intratumoral ^3^H-FLT uptake level significantly decreased at 6 hrs and then gradually increased with time in the radiation-treated groups (Figures 3, 4A, B). In the visual analysis of intratumoral ^18^F-FMISO and ^3^H-FLT ARG images, ^18^F-FMISO was distributed mostly in the central part of the tumor tissue, whereas ^3^H-FLT was distributed in the peripheral part of the tumor tissue in both the non-radiation-treated control and radiation-treated groups (Figure 3). In the radiation-treated groups, tumor growth was suppressed compared with that in the non-radiation-treated control group, but tumor volume in these mice gradually increased with time (Figure 2). The hypoxia fraction determined by pimonidazole IHC also showed no significant changes with time after single-dose radiotherapy among the non-radiation-treated control and radiation-treated groups (Figure 5A, B). The tumor proliferation marker, Ki-67 labeling index, did not show significant changes among the non-radiation-treated control and radiation-treated groups (Figure 6A, B). These findings indicate that radiation-induced intratumoral hypoxic and proliferative changes differed. These two factors showed different behaviors following radiotherapy until 7 days in the FaDu xenografts. Radiation-induced lethal/sublethal cellular damage followed by DNA repair and redistribution [22, 23] may underlie the dynamic changes in proliferation activity, whereas reoxygenation after a single-dose radiotherapy did not occur in FaDu xenografts.

Several studies have shown that reoxygenation of tumors occurs 4–10 hrs (early stage) and 1 or 2 days (late stage) after single-dose radiotherapy [3, 24, 25]. After radiotherapy, transient changes caused by cell death are likely to occur during the oxygenation of surviving cells. Owing to the increase in oxygen levels and therefore an increase in radiosensitivity of a subset of tumor cell population [3, 25]. Therefore in our study, first ^18^F-FMISO level was expected to decrease in concert with the attenuation of tumor hypoxia (%pimonidazole-positive area decrease) following radiotherapy as radiation-induced reoxygenation. Early detection of such changes may lead to the decrease in radiotherapy dose and total time in the fractionated radiotherapy schedule, which can reduce the undesirable side effects of radiotherapy. However, our data showed that tumor volume in the treated mice did not decrease following radiotherapy, and the intratumoral hypoxic state also did not change after the single dose of radiotherapy until 7 days. The hypoxia marker pimonidazole IHC also showed no significant changes among the non-radiation treated control and radiation-treated groups after radiotherapy. These findings may indicate that a single-dose 10- or 20-Gy radiotherapy has no effect on the hypoxic state in FaDu xenografts. FaDu cells are undifferentiated human hypopharyngeal squamous cell carcinoma cells that are characterized by moderate radiosensitivity [26, 27]. Kasten-Pisula et al. [28] showed that the apoptotic changes occurring in FaDu cells are very minimal and no changes were observed after a single dose of radiation over time. Less radiosensitivity and less apoptotic response to radiotherapy of the FaDu cells would be the possible reasons for the absence of changes in the hypoxia state after a single dose of radiotherapy until 7 days in our study. Changes in the tumor hypoxic state depend on many individualized tumor properties, including the number of cells affected by reoxygenation, the degree of oxygen increase, and the timing or progression of this effect during therapy [24, 25]. For these aggressive and less radiosensitive cell lines such as FaDu, further radiotherapy fractionation might induce reoxygenation. Petersen et al. [29] showed that significant reoxygenation (hypoxic changes) occurs after 12 days of fractionated radiotherapy (3 Gy/12f/12d = total 36 Gy) in FaDu xenografts. Another study [30] also showed that the mean pO_2_ (Oxylab pO_2_ system) significantly increased after 2 weeks of accelerated fractionated radiotherapy (total dose of 40 Gy). All these results also partially support the findings of our study.

On the other hand, the intratumoral ^3^H-FLT uptake level in this FaDu xenograft significantly decreased at an early time point (6 hrs) and then gradually but significantly increased with time, showing the dynamic proliferative changes as in our previous study [12]. FLT is a substrate of TK1, and FLT uptake positively correlates with cell growth and TK1 activity. The rapid decrease in FLT uptake level observed may be due to a rapid decrease in TK1 activity after radiation treatment [11, 14]. After radiotherapy, the strategy to deal with damaged DNA can be divided in to three components in eukaryotes: recognition of damaged DNA; a period of damage assessment; and the implementation of the appropriate response, namely, DNA repair and redistribution of cells among the cell-cycle phases or cell death [22]. These DNA repair and redistribution of cells among the cell-cycle phase might lead to cellular proliferation, which may increase TK1 activity and may be reflected by the increase in intratumoral FLT uptake level with time after a single dose of radiotherapy, as we discussed in our previous study [12]. In our study, we found the suppression of tumor growth after radiation treatment compared with the non-radiation-treated control group. However, there were no significant changes in the Ki-67 index among the non-radiation-treated control and radiation-treated groups in our study. Ki-67 is a nuclear protein and expressed during the late G1, S, G2, and M phases of the cell cycle. Radiotherapy causes G2/M phase arrest and the arrested cells during the cell cycle also contain Ki-67 even though they are not actively proliferating, as we discussed in our previous study [12].

Human head and neck tumors showed different hypoxic and proliferative patterns after radiotherapy, even when they have the same histological feature [31]. Thus, evaluation of dynamic changes in intratumoral hypoxic and proliferative states using the dual tracers FMISO and FLT is important. In our study, we used the less radiosensitive cell line FaDu and found no changes in intratumoral ^18^F-FMISO level. On the other hand, the intratumoral ^3^H-FLT uptake level significantly decreased at 6 hrs and then gradually increased with time. These findings indicate that single-dose 10- or 20-Gy radiotherapy may not affect the hypoxic state but still affect proliferative activity in this aggressive and less radiosensitive cell line. In our case, further radiotherapy fractionation may cause reoxygenation (decrease in ^18^F-FMISO level) and delay the gradual enhancement of proliferation. In this study, no significant hypoxic changes were found between 10- and 20-Gy radiation doses. Therefore, further radiotherapy fractionation (10 Gy or less) without dose escalation will be appropriate for this situation. If no marked changes occur after further radiotherapy fractionation, an immediate shift to a combination therapy or surgical procedures would be the treatment of choice. Thus, early evaluation of dynamic changes in intratumoral hypoxic and proliferative states by a dual-tracer study may provide important information for a better understanding of tumor biology after radiotherapy and for radiotherapy planning including dose escalation and altered fractionation schedules.

The limitation of our study was that only one tumor model, i.e., a moderately radiosensitive cancer cell line, was used to evaluate the dynamic changes in the tumor microenvironment (intratumoral hypoxic state and proliferative activity) after a single dose of radiation using dual tracers of ^18^F-FMISO and ^3^H-FLT. Various cancer cell lines, such as radiosensitive and radioresistant cancer cell lines should be used to confirm our present results.

## Conclusions

Our animal model showed no dynamic changes in intratumoral ^18^F-FMISO level after single-dose radiotherapy. On the other hand, ^3^H-FLT uptake level significantly decreased at 6 hrs and then gradually increased with time. These findings indicate that radiation-induced intratumoral hypoxic and proliferative changes differed and these two factors show different behaviors until 7 days in the FaDu xenografts. Thus, concomitant monitoring of dynamic changes in intratumoral hypoxic states and proliferative activity may provide important information for a better understanding of tumor biology after radiotherapy and for radiotherapy planning, including dose escalation and altered fractionation schedules.
